# EXPLORATION OF VARIABILITY IN STRESS AND SLEEP QUALITY IN OLDER PEOPLE WITH MILD COGNITIVE IMPAIRMENT

**DOI:** 10.1093/geroni/igae098.3970

**Published:** 2024-12-31

**Authors:** Sonoko Kabaya, Chieko Greiner, Yuko Yamaguchi, Masahide Nakamura

**Affiliations:** Kobe University, Kobe, Japan; Kobe University, Kobe, Japan; Kobe University, Kobe, Japan; Kobe University, Kobe, Japan

## Abstract

Mild cognitive impairment (MCI) is common, affecting 10%-35% of people over 65, since medical technology has advanced. Previous studies have reported that stress in older people with MCI exacerbates cognitive decline and can lead to daily depressive affect contagion between family caregivers (FCs) caring for older people with MCI. They have also indicated that stress adversely affects sleep quality and duration among older people with MCI. However, it is not clear how stress and sleep quality in older people with MCI fluctuate within a day especially throughout night. The aim of this study was to explore the characteristics of variability in stress and sleep quality in older people with MCI. We recruited dyads of older adults with MCI over the age of 65 and FCs. Data collection was performed continuously for 7 consecutive days. We adopted smart watches (GARMIN vívosmart 4) to measure Heart Rate Variability (HRV)-based stress and sleep quality. The Mann-Whitney U test was performed using Python 3.12. Results showed that stress levels were significantly higher during wakefulness compared to nighttime sleep (p=0.05e-89); however, no significant differences were observed based on the depth of sleep (p >.05). This finding is beneficial for considering how to reduce their stress especially during deep sleep and how to improve sleep quality.

